# Safety assessment of subchronic feeding of insect-resistant and herbicide-resistant transgenic soybeans to juvenile channel catfish (*Ictalurus punctatus*)

**DOI:** 10.1038/s41598-023-31072-2

**Published:** 2023-04-03

**Authors:** Dan Xiang, Mingzhong Luo, Fukun Jiang, Zhengrong Wen, Xiaoyun Chen, Xiaofu Wang, Xiaoli Xu, Wei Wei, Junfeng Xu

**Affiliations:** 1 State Key Laboratory for Managing Biotic and Chemical Threats to the Quality and Safety of Agro-Products, Institute of Agro-Product Safety and Nutrition, Zhejiang Academy of Agricultural Sciences, Hangzhou, 310021 China; 2 Engineering Research Centre of Ecology and Agricultural Use of Wetland, Ministry of Education, College of Animal Science, Yangtze University, Jingzhou, 434025 China; 3 Beijing DaBeiNong Biotechnology Co., Ltd., Beijing, 100193 China

**Keywords:** Enzymes, Genetic engineering, Animal physiology

## Abstract

Transgenic soybean is one of the most planted crops for human food and animal feed. The channel catfish (*Ictalurus punctatus*) is an important aquatic organism cultured worldwide. In this study, the effect of six different soybean diets containing: two transgenic soybeans expressing different types of *cp4-epsps*, *Vip3Aa* and *pat* genes (DBN9004 and DBN8002), their non-transgenic parent JACK, and three conventional soybean varieties (Dongsheng3, Dongsheng7, and Dongsheng9) was investigated in juvenile channel catfish for eight weeks, and a safety assessment was performed. During the experiment, no difference in survival rate was observed in six groups. The hepatosomatic index (HSI) and condition factor (CF) showed no significant difference. Moreover, comparable feed conversion (FC), feeding rate (FR), and feed conversion ratio (FCR) were found between transgenic soybean and JACK groups. Assessment of growth performance showed that the weight gain rate (WGR) and specific growth rate (SGR) of channel catfish were consistent. In addition, there were no changes in enzyme activity indexes (lactate dehydrogenase (LDH), total antioxidant capacity (T-AOC), aspartate aminotransferase (AST) and alanine aminotransferase (ALT)) in channel catfish among treatments. The research provided an experimental basis for the aquaculture feed industry to employ transgenic soybean DBN9004 and DBN8002 for commercial purposes.

## Introduction

Transgenic soybean is the most widely grown genetically modified (GM) crop^1^. In 2019, GM soybean planted on 91.9 million hectares, representing 48.27% of the world's total GM crop planted^2^. GM crops possess useful traits, such as herbicide tolerance, insect resistance, abiotic stress tolerance, disease resistance, and nutritional improvement^3^. With the widespread use of various GM crops worldwide, the potential associated risks should warrant attention^4^.

In recent years, a lot of research on the risk assessment of GM crops has essentially focused on terrestrial ecosystems. Importantly, it was found that crops with *Bacillus thuringiensis* (*Bt*) protein-coding genes would not harm non-target organisms such as bees^5^, but negatively impacted the survival and development of silkworms^6^. Similarly, transgenic *Bt* plants were documented to have little impact on the soil biota, including earthworms, collembolans, and the general soil microflora^7^. Furthermore, a 90-day rodent feeding experiment showed that phytase transgenic maize did not affect the health of mice^8^. Similarly, there was no significant difference in growth performance and feeding performance in rainbow trout (*Oncorhynchus mykiss*) fed transgenic defatted soybean meal for 12 weeks^9^. It is widely acknowledged that channel catfish is a warm-water fish that mainly feeds on benthos, small fish, shrimp, aquatic insect and organic waste. It is native to the rivers of North America, southern Canada and northern Mexico and is now cultured worldwide^10^. The channel catfish industry is important in the southern states of the United States and China^11^. In this regard, it represents the largest domestic aquaculture sector in the United States, with a yield reaching 15.8 billion kg in 2019^12^. The channel catfish is also a representative aquatic model animal in the American aquaculture environment.

Soybean is a valuable source of vegetable protein widely used in animal feed, mainly in ration formulations for farmed animal species (e.g., poultry, pigs, cattle and aquaculture)^13^. Importantly, higher quality and quantity of proteins have been reported in soybean seeds than in any other legumes^14^. Demand for GM soybeans and GM soybean meal has substantially increased in recent years accounting for a large share in revenues on the global market^15^. With increased land allocated to commercial cultivation of GM soybean, its application in aquatic feed processing line is bound to happen since it can also serve as a substitute for fish meal. Globally, up to 90% of the biomass of GM plants is used for animal feed purposes^16^. It has been reported that commercial GM soybeans, corn and other crops have been used by feed producers worldwide^17^. Feed is widely acknowledged as a significant source of waste in aquaculture systems, when the nutrients not absorbed are excreted into the water^18^, some GM products will enter aquatic systems as leached proteins^19^, which potentially results in direct uptake or some degree of exposure of different aquatic species to GM products and transgenes in the water^20^. Furthermore, as an important source of protein in animal feed, transgenic soybean can affect fish via their feed intake or the aquatic environment. For example, the DNA sequences from transgenic soybeans were found to survive through the gastrointestinal tract of Atlantic salmon fish^21^. Meanwhile, GM crop material has been found to enter aquatic environments and aquatic invertebrates^22^ resulting from debris of harvested GM plants released into streams^23^ or from GM corn entering water after open pollination^19^. The multiple herbicide-resistant transgenic soybean DBN9004 is a new genetically modified soybean event which is developed by DaBeiNong Biotechnology Co. Ltd and has obtained safety certificate in China in 2021. In 2022, another insect-resistant and herbicide-resistant transgenic soybean DBN8002 developed by the same company has obtained safety certificate in Argentina. Although many studies have evaluated the safety of GM soybeans as animal feed in aquatic animals, no study has assessed safety of GM soybeans DBN9004 and DBN8002 in channel catfish, to the best of our knowledge.

This study aimed to assess the somatic and biochemical effects of new insect-resistant and herbicide-resistant transgenic soybeans DBN9004 and DBN8002 in channel catfish fed for eight weeks.

## Materials and methods

### Sources of transgenic soybeans and feed processing

The multiple herbicide-resistant transgenic soybean DBN9004, insect-resistant and herbicide-resistant transgenic soybean DBN8002 and their non-transgenic parent JACK, and three conventional soybean varieties (Dongsheng3, Dongsheng7 and Dongsheng9) were used for channel catfish feeding experiments. The transgenic soybean DBN9004 (Code: DBN-Ø9ØØ4-6) was produced by inserting *cp4-epsps* and *pat* genes into soybean seed lines, and the transgenic soybean DBN8002 (Code: DBN-Ø8ØØ2-3) was produced by inserting *Vip3Aa* and *pat* genes. In this study, the non-transgenic parent JACK was served as a control, and three conventional soybean varieties were used to provide a range of variation for the non-transgenic and transgenic soybean varieties. All soybean varieties were provided from DaBeiNong Bioechnology Co., Ltd. (Beijing, China).

Before the experiment, all soybean varieties were processed into extruded full-fat soybean and made into granular feed with soybean meal inclusion level of 45%. In brief, each type of fish feed was crushed to powder and passed through a 80-mesh sieve and thoroughly mixed. The evenly mixed feed was pressed into pellets of 2.0 mm diameter using a granulator, then the feed pellets were air dried, packed with sealing bag and stored at room temperature. In this experiment, all six groups used the same culture conditions and the same proportion of feed formula, except for the different soybean varieties used in the feed (Table 1). Nutrient composition of six types of soybean feed was listed in Table 2.

**Table 1:** The composition and nutrient levels of the diet (Dry matter basis) %.

Items	Content (%)
Ingredients	
Fish meal	10
Corn gluten meal	6
Wheat flour	28
α-Cellulose	4.05
Anti-mould	0.1
Antioxidant	0.05
Ca(H_2_PO_4_)_2_	1.8
Soybean meal	45
Premix	5
Total	100.00
Nutrient levels	
Crude protein	32.90
Crude lipid	10.70
Total phosphorus	1.06
Met	0.50
Lys	1.69

**Table 2:** Nutrient composition of six types of soybean feed (${\overline{\text{X}}} \pm {\text{s}}$, %).

Species	Moisture	Crude protein	Crude lipid	Ca	P	Cys	Met	Lys
JACK	6.6	37.72	19.4	0.21	0.53	0.57	0.52	2.44
DBN9004	6.7	37.21	19.2	0.21	0.50	0.53	0.51	2.44
DBN8002	7.3	37.72	18.7	0.19	0.52	0.56	0.50	2.43
Dongsheng3	8.9	37.34	17.1	0.36	0.49	0.52	0.49	2.43
Dongsheng7	8.8	36.57	18.8	0.19	0.53	0.56	0.51	2.40
Dongsheng9	8.7	36.08	18.7	0.22	0.50	0.51	0.50	2.36

### Fish culture and experimental design

The experimental fish around three months of age were provided by a channel catfish breeding base (Xianning, Hubei, China). After the basic feed for the fish was domesticated for two weeks, channel catfish of similar size, average body weight of 3.15 ± 0.09 g and average body length of 5.84 ± 0.26 cm were selected for the next eight-week indoor experiment in the Aquatic Economic Animal Breeding Center of the College of Animal Science of Yangtze University. All experimental procedures were carried out in compliance with the regulations of the Guide for Care and Use of Laboratory Animals, which was approved by the Committee of Laboratory Animal Experimentation at Yangtze University. This study is reported in accordance with ARRIVE guidelines (Animal Research: Reporting of In Vivo Experiments).

Four hundred and eighty fish were randomly divided into 30 aquarium tanks, with 16 fish in each aquarium tank. Then, the 30 aquarium tanks were randomly arranged to six treatment groups (JACK (served as a control), DBN9004, DBN8002, Dongsheng3, Dongsheng7, and Dongsheng9), with five aquarium tanks (80 cm × 60 cm × 70 cm, length, width and height) in each group. During the experimental period, the channel catfish were fed the diets twice a day at 8:30 a.m. and 4:30 p.m. to apparent satiation, and the uneaten feed was removed by an aquarium vacuum cleaner 30 min after feeding. The food intake and number of fish death were recorded. During the experimental period, the fish were cultured in an aquarium recirculating system, and the water was exchanged 25% of the aquarium volume in every two days. The dissolved oxygen in the water was greater than 9.0 mg/L, the pH ranged of 7.21–7.81, the nitrite concentration was less than 0.05 mg/L, the ammonia nitrogen concentration was less than 0.02 mg/L, and the water temperature was maintained at 29.5–31.0 °C.

### Sample collection

At the end of the eight-week experimental trial, fifteen fish were randomly collected from each group and sacrificed. The liver, spleen or head kidney tissue samples were collected, and each type of tissue samples from five fish were pooled as one sample, and stored at −80 °C for enzyme activity analysis.

### Enzyme activity analysis measurement

The liver, spleen and head kidney tissues were weighed and homogenized with a ninefold volume of normal saline using a homogenizer. The lactate dehydrogenase (LDH), total antioxidant capacity (T-AOC), total protein (TP), aspartate aminotransferase (AST) and alanine aminotransferase (ALT) in the tissue homogenate were measured at 450 nm by a fluorescence microplate reader using commercial kits (NanJing JianCheng Bioengineering Institute, NanJing, Jiangsu, China).

### Evaluation of growth parameters performance

At the end of the eight-week experiment, the body length and weight were recorded from five fish per aquarium tank, with five tanks in each group (n = 25). The experimental fish were sacrificed, and the internal organs (e.g., heart, liver, stomach, intestine, kidney, spleen, gonad and swim bladder) were then weighed and recorded to calculate the growth parameters and morphological indices using the following Equations:

$$ \begin{aligned} & {\text{Survival rate }}({\text{SR}},\% ) = {\text{N}}_{{\text{t}}} /{\text{N}}_{0} \times {1}00\% \\ & {\text{Weight gain rate }}({\text{WGR}},\% ) = ({\text{W}}_{{\text{t}}} - {\text{W}}_{0} )/{\text{W}}_{0} \times {1}00\% \\ & {\text{Feed conversion ratio}}\;({\text{FCR}}) = {\text{W}}_{{\text{f}}} /({\text{W}}_{{\text{t}}} - {\text{ W}}_{0} ) \\ & {\text{Feeding rate}}\;{\text{(FR}},\% {)} = {\text{W}}_{{\text{f}}} /[({\text{W}}_{{\text{t}}} + {\text{ W}}_{0} ) \times {\text{t}}/{2}] \times {1}00\% \\ & {\text{Specific growth rate}}\;({\text{SGR}},\% /{\text{d}}) = ({\text{Ln }}({\text{W}}_{{\text{t}}} ) - {\text{Ln }}({\text{W}}_{0} ))/{\text{t}} \times {1}00 \\ & {\text{Hepatosomatic index }}({\text{HSI}},\% ) = {\text{W}}_{{\text{h}}} /{\text{W}} \times {1}00\% \\ & {\text{Viscerosomatic index }}({\text{VSI}},\% ) = {\text{W}}_{{\text{v}}} /{\text{W}} \times {1}00\% \\ & {\text{Condition factor }}({\text{CF}},{\text{g}}/{\text{cm}}^{{3}} ) = {\text{W}}/{\text{L}}^{{3}} \times {1}00 \\ \end{aligned} $$

N_t_ is the final number of channel catfish, N_0_ is the initial number, t is the feeding days (d), W_t_ is the final body weight (g), W_0_ is the initial body weight (g), W_f_ is the feed intake (g), Ln is the natural logarithm, W_h_ is the fish liver weight (g), W_v_ is the fish viscera weight (g), W is the fish body weight (g), and L is the length of fish (cm).

### Statistical analysis

The data were analyzed using Graphpad Prism 8 (GraphPad Software, San Diego, CA, USA). The survival rate was analyzed using a two-tailed Student's *t*-test. The body weight and body length were analyzed using one-way analysis of variance (ANOVA). The others were analyzed using two-way ANOVA. All data were expressed as mean ± standard error (S.E.). *p* values < 0.05 were considered statistically significant.

## Results

### Effects of transgenic soybeans on growth performance

The growth performance of channel catfish fed different diets was compared in Table 3. There was no significant difference in body weight and body length of channel catfish in the six groups at the end of the experiment. The WGR and SGR of the six groups ranged from 220.17 to 278.6% and from 2.07 to 2.37%/d, respectively, however, no significant difference was observed among the transgenic soybean DBN9004 and DBN8002 groups, JACK group and three conventional soybean groups.

**Table 3:** The effects on the growth performance of channel catfish fed transgenic soybeans (n = 25).

Items	Soybean type species						*P*
	JACK	DBN9004	DBN8002	Dongsheng3	Dongsheng7	Dongsheng9	
IBW (g)	3.19 ± 0.11	3.18 ± 0.07	3.12 ± 0.04	3.19 ± 0.06	3.04 ± 0.06	3.17 ± 0.06	0.140
FBW (g)	10.69 ± 1.44	10.18 ± 1.27	10.62 ± 0.39	12.08 ± 1.20	10.87 ± 1.11	10.51 ± 1.61	0.114
IBL (cm)	5.81 ± 0.30	5.80 ± 0.23	6.01 ± 0.33	5.83 ± 0.21	5.87 ± 0.26	5.80 ± 0.16	0.059
FBL (cm)	8.82 ± 0.41	8.77 ± 0.35	8.81 ± 0.06	9.22 ± 0.26	8.96 ± 0.41	8.86 ± 0.45	0.178
WGR (%)	235.07 ± 45.24	220.17 ± 39.83	240.44 ± 12.66	278.62 ± 37.74	257.61 ± 36.66	231.44 ± 50.75	0.123
SGR (%/d)	2.15 ± 0.23	2.07 ± 0.23	2.19 ± 0.07	2.37 ± 0.17	2.27 ± 0.19	2.12 ± 0.29	0.128

IBW, Initial body weight; FBW, final body weight; IBL, initial body length; FBL, final body length; WGR, weight gain rate; SGR, specific growth rate.

*p* < 0.05 indicates significant differences among the six experimental groups.

### Effects of transgenic soybean on survival and body indexes

**Figure 1:**
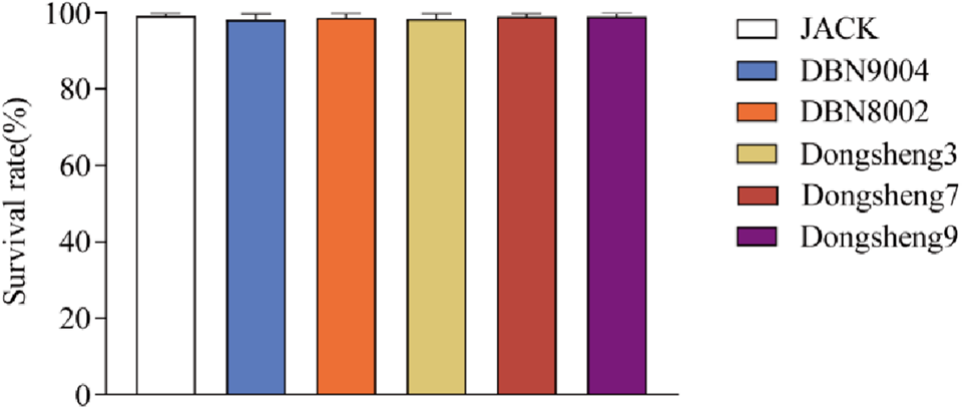
The survival rate of juvenile channel catfish fed different types of soybean diets. The total 30 aquarium tanks were randomly arranged to six treatment groups (JACK (served as a control), DBN9004, DBN8002, Dongsheng3, Dongsheng7, and Dongsheng9), with five aquarium tanks in each group. The survival rate of fish in each aquarium was recorded. Data are presented as mean ± SE (n = 5). *p < 0.05 *versus* JACK (*t*-test).

During the eight-week experiment, no abnormal behavior was observed in each group, only a few individual deaths occurred in the process of temporary culture (1 death in JACK group, 3 deaths in DBN9004 group, 2 deaths in DBN8002 group, 2 deaths in Dongsheng3 group, 1 death in Dongsheng7 group, and 1 death in Dongsheng9 group), and no significant difference in SR (ranging from 96.25 to 98.75%) was observed between two transgenic soybean groups and JACK group (Fig. 1). Assessment of body indices showed no significant difference in HSI (1.24–1.44%) and CF (1.50–1.54 g/cm^3^) among the groups (Table 4). The VSI of the transgenic soybean DBN9004 group (12.35%) was significantly different from the JACK group (11.11%), but still in the normal range of VSI among six groups (Table 4).

**Table 4:** The effects on the body index of channel catfish fed transgenic soybeans (n = 25).

Items	Soybean type species						*P*
	JACK	DBN9004	DBN8002	Dongsheng3	Dongsheng7	Dongsheng9	
HSI/%	1.44 ± 0.33	1.36 ± 0.08	1.24 ± 0.17	1.26 ± 0.13	1.32 ± 0.35	1.25 ± 0.08	0.864
VSI/%	11.11 ± 0.31^a^	12.35 ± 0.12^b^	11.38 ± 0.65^a^	11.72 ± 0.48^ab^	12.50 ± 0.56^b^	10.90 ± 0.20^a^	0.003
CF/g/cm^3^	1.51 ± 0.02	1.50 ± 0.03	1.54 ± 0.10	1.52 ± 0.03	1.50 ± 0.07	1.50 ± 0.01	0.909

HSI, Hepatosomatic index; VSI, viscerosomatic index; CF, condition factor.

*p* < 0.05 indicates significant differences among the six experimental groups.

### Effect of transgenic soybean on feeding

The FR and FCR of channel catfish fed six types of soybean feed were compared in Table 5. The results showed no significant difference in FR and FCR between two transgenic soybean groups and JACK (Table 5). Although the values of FCR in the DBN8002 group were higher than its non-transgenic counterpart parent JACK and three conventional soybean varieties, the difference was not statistically significant (*p* = 0.067).

**Table 5:** The effects on the feeding of channel catfish fed transgenic soybeans (n = 25).

Items	Soybean type species						*P*
	JACK	DBN9004	DBN8002	Dongsheng3	Dongsheng7	Dongsheng9	
FC (g)	16.21 ± 2.62	16.92 ± 1.60	18.76 ± 1.26	18.51 ± 0.67	17.48 ± 1.60	17.79 ± 1.80	0.476
FR (%)	4.16 ± 0.25	4.57 ± 0.84	4.88 ± 0.44	4.34 ± 0.19	4.53 ± 0.78	4.68 ± 0.65	0.657
FCR	2.16 ± 0.35	2.42 ± 0.23	2.50 ± 0.17	2.08 ± 0.08	2.23 ± 0.20	2.42 ± 0.25	0.067

FC, Feed conversion; FR, feeding rate; FCR, feed conversion ratio.

*p* < 0.05 indicates significant differences among the six experimental groups.

### Effect of transgenic soybean on enzyme activities

**Figure 2:**
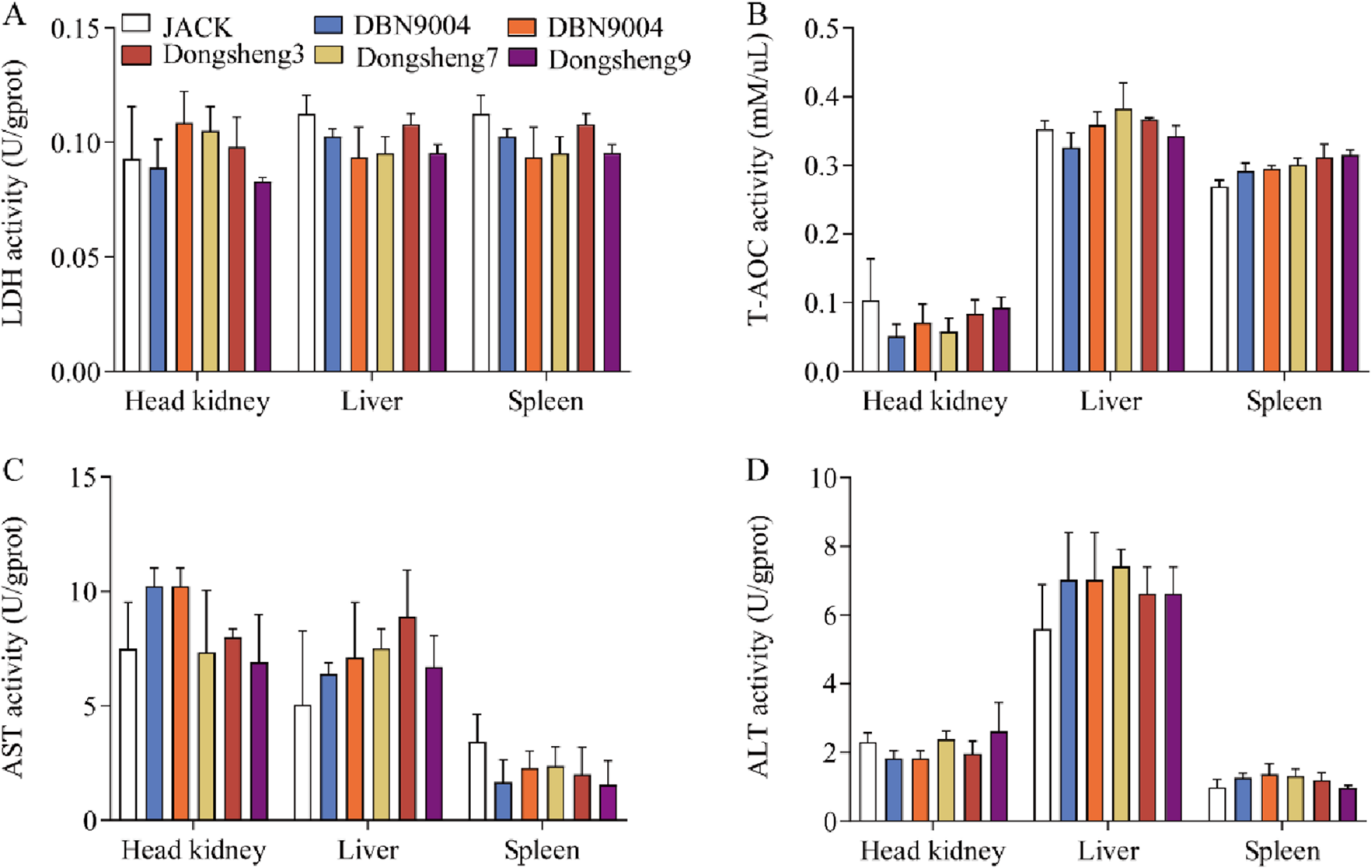
The enzyme activities of juvenile channel catfish fed different types of soybean diets. Head kidney, liver and spleen were collected from six groups of channel catfish at the end of the eight-week experiment, and (**A**) LDH, (**B**) T-AOC, (**C**) AST, (**D**) ALT were measured. The abscissa represents the sample type (Head kidney, Liver and Spleen) and different colors represent different sample groups. Data are presented as mean ± SE (n = 3). *p < 0.05 versus JACK (*t*-test).

The effect of GM soybean on the head kidney, liver and spleen of channel catfish LDH, T-AOC, AST and ALT enzyme activity is shown in Fig. 2. After the eight-week feeding experiment, no differences of enzyme activity were observed among transgenic soybean DBN9004, DBN8002 and JACK groups, and Analyses of tissue samples head kidney, liver, and spleen from target organs were found that no visible variation in treatment groups.

## Discussion

In our study, we assess the risk of two new transgenic soybean as the main ingredient for channel catfish diets. According to our knowledge, this is the first study in channel catfish that has assessed the safety of transgenic soybeans containing *cp4-epsps*, *Vip3Aa* and *pat* genes over such an extended period of time.

Channel catfish is a valuable species that serve as an ideal model for physiological studies because of its ecological and economical importance^24^. The embryo or juvenile stage is the most important period in the life cycle, especially sensitive to environmental toxins^25^. In this respect, Kennedy et al. reported that the juvenile mussel (*Mytilus chilensis*) was particularly sensitive to ammonia^26^. Moreover, Kim et al. found dietary chromium exposure to juvenile rockfish could induce significant chromium accumulation in the specific tissues, inhibit growth, and cause hematological alterations^27^. Accordingly, we used juvenile channel catfish to assess the safety of two transgenic soybeans, DBN9004 and DBN8002, in terms of survival, growth performance, feeding rate, body index, and enzymatic activity.

After eight weeks feeding, the results showed that there were no significant differences in survival, growth performance, feeding rate, body index, and enzymatic activities between two transgenic soybean groups and the parental JACK group. During the experiment, the survival and growth performance (e.g., initial body weight, final body weight, initial body length, final body length, weight gain rate, and specific growth rate) showed no differences between two transgenic soybean groups and the JACK group. Consistent to our findings, Sanden et al. reported that there were no differences in growth of Atlantic salmon fed with GM soybean and control diets for six weeks^21^. Similarly, Suharman et al. reported that no harmful effects were observed on the survival rate of juvenile common carp fed with GM soybean meal in comparison to non-GM soybean meal^28^. The feeding study of two lines of glyphosate-tolerant soybeans fed to rats, broiler chickens, catfish and dairy cows showed no adverse effect to these animals^29^. Recently, a long-term oral toxicity study was carried out for the safety assessment of transgenic rice containing Cry1Ab protein in the highly inbred Wuzhishan pigs, and no significant difference on the growth, reproductive performance, hematology, or organ morphology were found between GM and non-GM groups^30^, and these results indicated that transgenic soybean is as safe as conventional non-GM soybean. However, the body index of our researches showed that the VSI of channel catfish fed with transgenic soybean DBN9004 (12.35 ± 0.12) were higher than that in JACK (11.11 ± 0.31), but still in the normal range from 10.90 to 12.50 in conventional soybean groups. The higher VSI indicated better growth of the fish fed with transgenic soybean DBN9004. The similar nutrient composition of six types of soybean feed has been tested, so it suggested that the main cause of differences in the growth performance may be in feed intake caused by organoleptic properties, rather than in nutrient composition. Similarly, Sissener et al. reported that zebrafish fed with GM maize exhibited significantly better growth than that fed non-GM group^31^. Sanden et al. found the offspring zebrafish fed the *Bt* (*Bacillus thuringiensis*) maize exhibited enhanced growth performance than those fed non-*Bt* maize, and speculated that this may be related to reduced mycotoxin levels in *Bt* maize^32^. Mycotoxins were highly contaminated in fish feed^33^, and it was reported that *Bt* corn has significantly lower aflatoxin levels than non-*Bt* corn^34^. So the transgenic soybeans may reduce the mycotoxins induced reduction of growth and health status of fish. Therefore, we thought the new transgenic soybeans are as safe as non-GM soybean, although the cause for the difference of growth performance is not clear.

It is widely acknowledged that LDH is an important enzyme in animal cells^35^, and commonly used in environmental physiology and disease diagnosis and analysis^36^. T-AOC is a comprehensive index used to measure the functional status of the enzymatic and non-enzymatic components of the antioxidant system^37^. A study indicated that bacterial infection (e.g., *Aeromonas hydrophila*) decreased the activity of T-AOC in common carp^38^. In this study, although no significant difference of T-AOC was observed among transgenic soybean DBN9004, DBN8002 groups and JACK group, the lower level of T-AOC in head kidney of DBN9004 group indicated that the antioxidative defense system may be impaired to a certain extent and this result may be caused accidentally by bacterial infection during the eight-week experiment or in some instances be related to sampling error. AST and ALT have been reported to be sensitive indicators that can reflect the function of hepatocytes and protein metabolism of fish^39^. The increases in AST and ALT activities are considered as a result of liver damage by toxicants. In this study, although no significant differences of AST and ALT was observed among transgenic soybean DBN9004, DBN8002 groups and JACK group, the higher level of AST and ALT in liver of DBN9004 and DBN8002 groups indicated that the function of hepatocytes may be influenced by the bacterial toxins or be related to sampling error. In the present study, no significant difference in enzymatic activities was found in the head kidney, liver and spleen of channel catfish fed with transgenic soybeans and JACK, consistent to the findings by Gao et al. and Magaña-Gómez et al., which documented that the plasma amylase level of mice fed with transgenic soybean and the activities of SOD, malondialdehyde (MDA) and CAT of zebrafish larvae exposed to two *Bt* proteins were unaltered, respectively^40,41^. Moreover, a similar finding was reported by Sanden et al. that no significant difference of AST and ALT in plasma of Atlantic salmon fed GM corn and conventional soybean for eight months^42^.

In conclusion, during the eight-week experiment, a high survival and normal growth status were observed in channel catfish. No significant difference was found between transgenic soybeans (DBN9004 and DBN8002) and JACK in terms of survival, body index (HSI, CF, except VSI), food intake (FC, FR and FCR), growth performance (WGR and SGR) and enzymatic activity (LDH, T-AOC, AST and ALT), which indicated that transgenic soybeans DBN9004 and DBN8002 had the same safety profile as non-transgenic soybean.

## Data Availability

The datasets used and/or analysed during the current study available from the corresponding author on reasonable request.

## References

[1] James C (2018). Brief 54: Global Status of Commercialized Biotech/GM Crops.

[2] International Service for the Acquisition of Agri-biotech Applications (ISAAA). *Global Status of Commercialized Biotech/GM Crops in 2019: Biotech Crops Drive Socio-economic Development and Sustainable Environment in the New Frontier*. (ISAAA, 2019).

[3] Kumar K (2020). Genetically modified crops: Current status and future prospects. Planta.

[4] Gilbert N (2013). Case studies: A hard look at GM crops. Nature.

[5] Tang X (2020). Chronic toxicity study in Sprague-Dawley rats on transgenic rice T1c–19 with cry1C* gene. Food Chem. Toxicol..

[6] Yao HW, Jiang CY, Ye GY, Hu C, Peng YF (2008). Toxicological assessment of pollen from different Bt rice lines on *Bombyx mori* (Lepidoptera: Bombyxidae). Environ. Entomol..

[7] O'Callaghan M, Glare TR, Burgess EP, Malone LA (2005). Effects of plants genetically modified for insect resistance on nontarget organisms. Annu. Rev. Entomol..

[8] Liang C (2021). Safety assessment of phytase transgenic maize 11TPY001 by 90-day feeding study in rats. Food Chem. Toxicol..

[9] Chainark P (2006). Availability of genetically modified soybean meal in rainbow trout *Oncorhynchus mykiss* diets. Fish. Sci..

[10] Limbaugh N (2021). Coping strategies in response to different levels of elevated water hardness in channel catfish (*Ictalurus punctatus*): Insight into ion-regulatory and histopathological modulations. Comp. Biochem. Physiol. A Mol. Integr. Physiol..

[11] Zhong L (2016). Channel catfish in China: Historical aspects, current status, and problems. Aquaculture.

[12] Potter N (2019). rnassqs: An R package to access agricultural data via the USDA National Agricultural Statistics Service (USDA-NASS) 'Quick Stats' API. J. Stat. Softw..

[13] EFSA Panel on Genetically Modified Organisms (GMO) et al*.* Assessment of genetically modified soybean MON 87705 × MON 87708 × MON 89788, for food and feed uses, under regulation (EC) No 1829/2003 (application EFSA-GMO-NL-2015-126). *EFSA J.* **18**, e06111 (2020). 10.2903/j.efsa.2020.6111 PMC10464710 37649527

[14] Natarajan S, Luthria D, Bae H, Lakshman D, Mitra A (2013). Transgenic soybeans and soybean protein analysis: An overview. J. Agric. Food Chem..

[15] Tillie, P. & Rodríguez-Cerezo, E. Markets for non-genetically modified, identity-preserved soybean in the EU (2015).

[16] Damaziak K (2021). Effects of replacement genetically modified soybean meal by a mixture of: Linseed cake, sunflower cake, guar meal and linseed oil in laying hens diet. Production results and eggs quality. Anim. Feed Sci. Technol..

[17] Bakke-McKellep AM (2007). Histological, digestive, metabolic, hormonal and some immune factor responses in Atlantic salmon, *Salmo salar* L., fed genetically modified soybeans. J. Fish. Dis..

[18] Eya JC, Ashame MF, Pomeroy CF (2010). Influence of diet on mitochondrial complex activity in channel catfish. N. Am. J. Aquac..

[19] Carstens K (2012). Genetically modified crops and aquatic ecosystems: Considerations for environmental risk assessment and non-target organism testing. Transgenic Res..

[20] Hilbeck A (2017). Procedure to select test organisms for environmental risk assessment of genetically modified crops in aquatic systems. Integr. Environ. Assess. Manag..

[21] Sanden M, Bruce IJ, Rahman MA, Hemre GI (2004). The fate of transgenic sequences present in genetically modified plant products in fish feed, investigating the survival of GM soybean DNA fragments during feeding trials in Atlantic salmon *Salmo salar* L. Aquaculture.

[22] Parrott W (2008). Study of Bt impact on caddisflies overstates its conclusions: Response to Rosi-Marshall et al.. Proc Natl Acad Sci USA.

[23] Chen Y, Romeis J, Meissle M (2021). Performance of *Daphnia magna* on flour, leaves, and pollen from different maize lines: Implications for risk assessment of genetically engineered crops. Ecotoxicol. Environ. Saf..

[24] Stewart HA, Aboagye DL, Ramee SW, Allen PJ (2019). Effects of acute thermal stress on acid–base regulation, haematology, ion-osmoregulation and aerobic metabolism in Channel Catfish (*Ictalurus punctatus*). Aquac. Res..

[25] Crane M, Maltby L (1991). The lethal and sublethal responses of gammarus pulex to stress: Sensitivity and sources of variation in an in situ bioassay. Environ. Toxicol. Chem..

[26] Kennedy AJ, Lindsay JH, Biedenbach JM, Harmon AR (2017). Life stage sensitivity of the marine mussel *Mytilus edulis* to ammonia. Environ. Toxicol. Chem..

[27] Kim JH, Kang JC (2016). The chromium accumulation and its physiological effects in juvenile rockfish, *Sebastes schlegelii*, exposed to different levels of dietary chromium (Cr(6+)) concentrations. Environ. Toxicol. Pharmacol..

[28] Suharman I (2010). Suitability of genetically modified soybean meal in a dietary ingredient for common carp *Cyprinus carpio*. Fish. Sci..

[29] Hammond BG (1996). The feeding value of soybeans fed to rats, chickens, catfish and dairy cattle is not altered by genetic incorporation of glyphosate tolerance. J. Nutr..

[30] Liu Q (2018). Effects of long-term feeding with genetically modified Bt rice on the growth and reproductive performance in highly inbred Wuzhishan pigs. Food Control.

[31] Sissener NH (2010). Zebrafish (*Danio rerio*) as a model for investigating the safety of GM feed ingredients (soya and maize); performance, stress response and uptake of dietary DNA sequences. Br. J. Nutr..

[32] Sanden M (2013). Cross-generational feeding of Bt (*Bacillus thuringiensis*)-maize to zebrafish (*Danio rerio*) showed no adverse effects on the parental or offspring generations. Br. J. Nutr..

[33] Nogueira WV (2020). Occurrence and bioacessibility of mycotoxins in fish feed. Food Addit. Contam. Part B.

[34] Franzosa EA (2019). Gut microbiome structure and metabolic activity in inflammatory bowel disease. Nat. Microbiol..

[35] Hou, X. M., Yuan, S. Q., Zhao, D., Liu, X. J. & Wu, X. A. LDH-A promotes malignant behavior via activation of epithelial-to-mesenchymal transition in lung adenocarcinoma. *Biosci. Rep.* **39** (2019). 10.1042/BSR20181476 PMC6331674 30509961

[36] Philipp DP, Parker HR, Whitt GS (1983). Evolution of gene regulation: Isozymic analysis of patterns of gene expression during hybrid fish development. Isozymes Curr. Top. Biol. Med. Res..

[37] Wang J (2021). The probiotic properties of different preparations using Lactococcus lactis Z-2 on intestinal tract, blood and hepatopancreas in Cyprinus carpio. Aquaculture.

[38] Trivedi SP, Ratn A, Awasthi Y, Kumar M, Trivedi A (2021). In vivo assessment of dichlorvos induced histological and biochemical impairments coupled with expression of p53 responsive apoptotic genes in the liver and kidney of fish, Channa punctatus (Bloch, 1793). Comp. Biochem. Physiol. C Toxicol. Pharmacol..

[39] Vijayavel K, Balasubramanian MP (2006). Fluctuations of biochemical constituents and marker enzymes as a consequence of naphthalene toxicity in the edible estuarine crab *Scylla serrata*. Ecotoxicol. Environ. Saf..

[40] Gao YJ (2018). Safety Assessment of *Bacillus thuringiensis* insecticidal proteins Cry1C and Cry2A with a zebrafish embryotoxicity test. J. Agric. Food Chem..

[41] Magana-Gomez JA, Cervantes GL, Yepiz-Plascencia G, de la Barca AM (2008). Pancreatic response of rats fed genetically modified soybean. J. Appl. Toxicol..

[42] Sanden M, Krogdahl A, Bakke-Mckellep AM, Buddington RK, Hemre GI (2006). Growth performance and organ development in Atlantic salmon, *Salmo salar* L. parr fed genetically modified (GM) soybean and maize. Aquac. Nutr..

